# Residential Green Space and Cognitive Function in a Large Cohort of Middle-Aged Women

**DOI:** 10.1001/jamanetworkopen.2022.9306

**Published:** 2022-04-27

**Authors:** Marcia P. Jimenez, Elise G. Elliott, Nicole V. DeVille, Francine Laden, Jaime E. Hart, Jennifer Weuve, Francine Grodstein, Peter James

**Affiliations:** 1 Department of Epidemiology, Harvard T.H. Chan School of Public Health, Boston, Massachusetts; 2 Now with Department of Epidemiology, Boston University School of Public Health, Boston, Massachusetts; 3 Department of Environmental Health, Harvard T.H. Chan School of Public Health, Boston, Massachusetts; 4 Channing Division of Network Medicine, Department of Medicine, Brigham and Women’s Hospital, Harvard Medical School, Boston, Massachusetts; 5 Department of Epidemiology, Boston University School of Public Health, Boston, Massachusetts; 6 Department of Internal Medicine, Rush Medical College, Rush Alzheimer’s Disease Center, Chicago, Illinois; 7 Department of Population Medicine, Harvard Medical School, Harvard Pilgrim Health Care Institute, Boston, Massachusetts

## Abstract

**Importance:**

Green space can decelerate cognitive decline by supporting physical activity, psychological restoration, or reducing exposure to air pollution. However, existing studies on the association of green space with cognitive decline are limited.

**Objective:**

To examine whether residential green space was associated with cognitive function in middle-aged women.

**Design, Setting, and Participants:**

Starting in 1989, the Nurses’ Health Study II enrolled 116 429 female nurses aged 25 to 42 years residing in the US. In 2014 to 2016, 40 082 women were invited to complete an online cognitive battery. This cohort study analyzed women who had data on both green space exposure and cognitive measures. Data analysis was conducted from June to October 2021.

**Exposures:**

Residential exposure to green space was assessed using the Normalized Difference Vegetation Index, a satellite-derived indicator of the quantity of ground vegetation. Landsat satellite data at 270-m and 1230-m buffers around each participants’ residential addresses in 2013 were used.

**Main Outcomes and Measures:**

In 2014 to 2016, cognitive function was measured using a self-administered online battery, the Cogstate Brief Battery, consisting of 4 tasks measuring psychomotor speed, attention, learning, and working memory; 3 composite scores, averaging together all tasks, psychomotor speed/attention, and learning/working memory, were created. In addition, the study evaluated potential mediators, including air pollution, depression, and physical activity.

**Results:**

The analytical sample included 13 594 women, of whom 13 293 (98%) were White. Mean (SD) age was 61.2 (4.6) years. In models adjusted for age at assessment, race, childhood, adulthood, and neighborhood socioeconomic status, green space was associated with higher scores on the global Cogstate composite (mean difference per IQR in green space, 0.05; 95% CI, 0.02 to 0.07), and psychomotor speed/attention (mean difference in score, 0.05 standard units; 95% CI, 0.02 to 0.08). This difference in scores is similar to the difference observed in women 1 year apart in age in the data. By contrast, there was no association between green space and learning/working memory (mean difference, 0.0300; 95% CI, −0.0003 to 0.0500).

**Conclusions and Relevance:**

These findings suggest that increasing residential green space may be associated with modest benefits in cognition in middle-aged women.

## Key Points

**Question:**

Is there an association between exposure to green space and cognitive function throughout middle age?

**Findings:**

In this cohort study that included 13 594 women, increasing green space was associated with higher scores of overall cognition and psychomotor speed/attention. In contrast, there was no association between green space and learning/working memory.

**Meaning:**

These findings suggest that green space exposure should be investigated as a potential population-level approach to improve cognitive function.

## Teaser

This cohort study evaluates the association between residential green space and cognitive function in middle-aged women using data from the Nurses’ Health Study II.

## Introduction

The rising prevalence of Alzheimer Disease and related dementias calls for novel prevention strategies. Cognitive function at middle age is associated with subsequent dementia^1,2,3^ and is considered a risk factor for decline in physical functioning^4^ and mortality.^5^ Recent studies^6,7^ show that higher residential surrounding green space is associated with improved cognitive function in older adults. However, existing studies on the association of green space and cognitive function are still scarce.

Exposure to green space may be associated with cognitive function through increasing opportunities for physical activity, social engagement, psychological restoration, improving cognitive capacity, and mitigating the negative consequences of noise and air pollution.^8,9,10^ Although physical activity has been shown to decrease dementia risk,^11^ poor social engagement has been associated with increased dementia risk.^12^ The association between psychological restoration and dementia has been documented across population-based studies.^13,14^ In this research, the attention restoration theory states that people can recover from stress and mental fatigue and can concentrate better after spending time in or even looking at green space.^15^ Finally, there is increasing evidence that higher exposure to air pollution and noise are associated with faster cognitive decline^16,17,18,19,20^ and higher dementia prevalence.^21^

We used data from a nationwide prospective cohort study of women to investigate the associations between residential green space and cognitive function. Our green space exposure temporally preceded the cognitive function measures, and we evaluated potential mediators that also temporally preceded cognitive function measurement, which provide a step forward to advancing this area of research.^22^ We assessed effect modification by measures of neighborhood socioeconomic status (SES) and urbanicity.^23^ We hypothesized that green space exposure was positively associated with cognitive function in a nationwide prospective cohort study of women. Additionally, we hypothesized that this association would be most visible among women with lower neighborhood SES, and that the association would be mediated by depression, air pollution, and physical activity.

## Methods

### Participants

Starting in 1989, the Nurses’ Health Study II enrolled 116 429 female nurses aged 25 to 42 years residing in the US.^24^ Women complete mailed or online questionnaires biennially and follow-up is ongoing.^25^ Participants’ residential addresses throughout follow-up have been geocoded to obtain latitude and longitude. Between 2014 to 2016, 40 082 women who had completed a supplemental violence questionnaire, and with known email addresses, were invited to complete an online cognitive battery.^26^ Of those, 14 151 women completed the Cogstate Brief Battery,^27^ a self-administered computerized cognitive assessment, and 13 994 had complete data for all Cogstate scores. Responders to the invitation to complete the Cognitive Study were comparable to the nonresponders in terms of sociodemographics, depression, health behaviors, and medical conditions potentially relevant to cognitive function.^28^ The study protocol was approved by the institutional review board of the Brigham and Women’s Hospital. Return of the questionnaires and Cogstate completion constituted consent. We included data from women who had data on both green space and cognitive measures. This study follows the Strengthening the Reporting of Observational Studies in Epidemiology (STROBE) reporting guideline.^29^

### Measures

#### Green Space

Exposure to green space around participants’ home addresses in 2013 was estimated using the Normalized Difference Vegetation Index (NDVI), a satellite image–based metric (further details in the eAppendix in the Supplement). Briefly, NDVI ranges between −1 and 1, where negative values correspond to water, values around 0 correspond to barren areas of rock or sand, and positive values represent grassland. We examined the average NDVI in 270-m and 1230-m buffers around each geocoded address to evaluate both the immediate area around residences and the walkable area as potential relevant geographic contexts.^30^ NDVI was assessed as a continuous variable defined by the IQR for analysis. Negative and missing NDVI values (400 missing [2.9%]) were set to 0 using the rationale that green space is only captured on the positive scale.

#### Cognitive Function

The Cogstate Battery has 4 tasks: Detection, Identification, One Card Learning, and One Back.^27,31^ A detailed description of each task can be found in the eAppendix in the Supplement. The validity of Cogstate has been established in previous studies^27,32,33^ and has been shown to be a sensitive indicator of early cognitive deficits^32^ and Alzheimer dementia.^34,35^ On the basis of previous research,^28,36^ we created 3 composite scores: (1) a psychomotor speed/attention composite (created by averaging *z* scores of Detection and Identification, with a possible range of −6.9 to 3.4), (2) a learning/working memory composite (averaging *z* scores from One Card Learning and One Back, with a possible range of −5.9 to 3.8), and (3) an overall cognition composite (averaging *z* scores from all 4 tasks, with a possible range of −6.4 to 4.0).^31^ The 3 composite scores were standardized to have a mean of 0 and an SD of 1; all composite scores have had high test-retest reliability and clinical utility in identifying cognitive impairment.^34^ Higher scores indicate better cognitive performance.

#### Covariates

We selected covariates that are associated with green space and have been associated with cognitive function, but that were unlikely to result from either,^37^ including age at cognitive assessment and self-identified race (White, Black, or Asian). We adjusted for race to capture the outcomes of perceived race, along with various other factors such as quality of schools that are associated with skin color, genetic background, and cultural context.^38^ Additional covariates assessed in 2005 measuring childhood SES included maternal and paternal education level (percentage with less than a high school education), parents’ occupation (percentage who were unskilled laborers), and parents’ home ownership (yes/no). Covariates measured during participant’s adulthood, closest to the cognitive assessment, included individual and neighborhood SES. Individual SES was measured using 2 variables: husband’s education assessed in 1999 (less than high school, high school, 2 years of college, 4 years of college, or graduate school), and marital status (yes/no) assessed in 2013. Neighborhood SES was assessed by 2 variables: 2010 US Census tract median income and median home value based on the US Census tract of residence in 2013.

#### Effect Modifiers

On the basis of previous research, US Census tract median income and urbanicity were used as stratifiying variables.^23^ Urbanicity was measured as the US Census tract population density (number per square kilometers).

#### Mediators

Green space has been associated with decreased levels of air pollution,^39,40^ lower risk of depression, and increased physical activity,^40^ which, in turn, could improve cognitive function.^16,41,42,43^ Thus, we used mediation analysis^44,45,46^ to evaluate whether the association between green space and cognition is mediated through these variables of interest (eFigure 1 in the Supplement). We used spatiotemporal smoothing models to estimate exposures to particulate matter with a diameter less than 2.5 μm (eAppendix in the Supplement).^47^ Depressive symptoms were assessed in 2013 with the Center for Epidemiologic Studies Depression Scale and modeled as a continuous variable. Finally, physical activity was calculated from self-reported time spent per week (<3, 3 to <9, 9 to <18, 18 to <27, or ≥27 metabolic equivalent hours per week) in 2013.

### Statistical Analysis

We used multivariable linear regression to estimate differences in cognitive function across levels of green space exposure. Separate models were conducted for each cognitive composite score. We estimated mean differences (and 95% CIs) in cognitive composites corresponding to a 1-IQR increment in average NDVI in the 270-m buffer for our main results, and in the 1230-m buffer for sensitivity analyses. For each cognitive composite, we initially adjusted for age and race (model 1). Model 2 further adjusted for maternal and paternal education level, parents’ occupation, and parents’ home ownership. Model 3 further adjusted for husband’s education and marital status. Model 4 further adjusted for US Census tract median income and median home value. Natural splines were used to examine nonlinear associations of green space with cognitive function. To assess deviations from linearity for associations with all cognition measurements, we fit generalized additive models for continuous exposures and natural splines with 3 to 4 knots based on Akaike Information Criterion.

We assessed effect modification of associations of green space with cognitive function by Census tract median household income, and urbanicity using stratified analysis, multiplicative interaction terms and likelihood ratio tests to assess significance. We used mediation analysis^44,45,46^ to evaluate whether the outcome associated with green space is mediated through air pollution, depression, and physical activity, on each of the cognitive composites (eAppendix in the Supplement). Finally, as a sensitivity analysis, to address the fact that the mediators of interest could also be confounders, we further adjusted model 4 for air pollution, depression, and physical activity. Data analysis was conducted in SAS statistical software version 9.4 (SAS Institute) from June to October 2021. A 2-tailed t-test was used to calculate *P* values, and < .05 was considered significant.

## Results

Of 13 594 total participants (mean [SD] age of 61.2 [4.6] years), virtually all were White (13 293 participants [98%]). Women who lived in areas with higher levels of green space were less likely to be African American, more likely to have parents who had owned their home, and more likely to be married (Table 1). Areas with higher levels of green space were less likely to be densely populated (Table 1).

**Table 1.** Descriptive Statistics by Increasing Quintiles of Green Space in 2013 (270-m Buffer), Nurses’ Health Study II

Characteristic	Participants, No. (%) (N = 13 594)				
	Quintile 1: <0.34 (n = 2716)	Quintile 2: 0.34-0.46 (n = 2718)	Quintile 3: 0.47-0.54 (n = 2720)	Quintile 4: 0.55-0.62 (n = 2721)	Quintile 5: >0.62 (n = 2719)
Age at cognitive assessment, mean (SD), y	61.7 (4.4)	61.3 (4.6)	61.1 (4.6)	60.9 (4.7)	60.9 (4.6)
Race					
African American	26 (1.0)	18 (0.7)	17 (0.6)	10 (0.4)	8 (0.3)
White	2590 (95.4)	2646 (97.4)	2681 (98.6)	2693 (99.0)	2683 (98.7)
Parental education					
Mother, less than high school	463 (17.0)	442 (16.3)	488 (17.9)	491 (18.0)	504 (18.5)
Father, less than high school	630 (23.2)	659 (24.2)	675 (24.8)	653 (24.0)	711 (26.1)
Parental occupation, unskilled laborer	232 (8.5)	267 (9.8)	285 (10.5)	321 (11.8)	343 (12.6)
Parents owned home	1344 (49.5)	1339 (49.3)	1368 (50.3)	1398 (51.4)	1417 (52.1)
Husband’s education, <4 y college, 1999	718 (26.4)	749 (27.6)	826 (30.4)	901 (33.1)	989 (36.4)
Currently married, 2013	1884 (69.4)	2037 (74.9)	2082 (76.5)	2212 (81.3)	2310 (85.0)
Neighborhood median income (2010 US Census) in 2013, mean (SD), $	89 377 (34 615.1)	85 479.3 (34 109.1)	80 912.9 (29 795.6)	81 484.8 (29 010.6)	80 706.4 (30 387.3)
Neighborhood median home value (2010 US Census) in 2013, mean (SD), $	439 127.5 (254 109.9)	293 213.2 (220 267.7)	227 986.3 (152 116.5)	224 769.2 (141 436)	236 081.9 (155 451.6)
Population density, (2010 US Census) in 2013, mean (SD), No. of individuals/km^2^	2735.8 (5792.5)	1234.2 (1955.8)	813.1 (779.4)	546.7 (555.1)	235.5 (322.6)
Air pollution PM_2.5_, 2013, mean (SD), μg/m^3^	11.2 (3)	11.1 (2.3)	11.8 (2.1)	11.6 (2.1)	10.6 (2.2)
Depression score (CESD) in 2013, mean (SD)	6.0 (4.9)	5.8 (4.9)	5.8 (4.9)	5.7 (4.8)	5.5 (4.6)
Physical activity, 2013, metabolic equivalent hours/wk					
<3	274 (10.1)	263 (9.7)	281 (10.3)	286 (10.5)	261 (9.6)
3-8.9	409 (15.1)	430 (15.8)	422 (15.5)	420 (15.4)	415 (15.3)
9-17.9	476 (17.5)	561 (20.6)	552 (20.3)	544 (20.0)	518 (19.1)
18-26.9	408 (15.0)	344 (12.7)	390 (14.3)	394 (14.5)	385 (14.2)
Learning/working memory *z* score, mean (SD)	−0.03 (1.0)	−0.02 (1.0)	−0.01 (1.0)	0.03 (1.0)	0.04 (1.0)
Psychomotor speed/attention *z* score, mean (SD)	−0.08 (1.0)	−0.01 (1.0)	−0.002 (1.0)	0.04 (1.0)	0.05 (0.9)
Global Cogstate *z* score, mean (SD)	−0.07 (1.0)	−0.02 (1.0)	−0.01 (1.0)	0.04 (1.0)	0.06 (0.9)

Abbreviations: CESD, Center for Epidemiologic Studies Depression Scale; PM_2.5_, fine particulate matter.

In models adjusted for age and race, 1 IQR higher green space exposure in a 270-m buffer was associated with higher scores on the psychomotor speed/attention composite (β, 0.04; 95% CI, 0.02 to 0.07) (Table 2) and on overall cognition (β, 0.04; 95% CI, 0.01 to 0.06) (Table 2). Further adjustment for individual childhood and adulthood SES (β, 0.05; 95% CI, 0.02 to 0.08) and neighborhood SES (β, 0.05; 95% CI, 0.02 to 0.07) did not attenuate these associations, and the results remained virtually unchanged. Similarly, in sensitivity analyses using the 1230-m buffer, in fully adjusted models, 1 IQR higher of green space exposure was associated with higher scores on the psychomotor speed/attention composite (β, 0.04; 95% CI, 0.02 to 0.07) (eTable 1 in the Supplement) and on overall cognition (β, 0.04; 95% CI, 0.02 to 0.06) (eTable 1 in the Supplement). In the main analyses within a 270-m buffer and in sensistivity analyses within a 1230-m buffer, green space exposure was not associated with higher scores on the learning/working memory composite (fully adjusted within 270-m buffer: β, 0.03; 95% CI, −0.000 to 0.05; 1230-m buffer: β, 0.02; 95% CI, −0.01 to 0.04). The estimate of the association between green space and processing speed and attention was equivalent to the estimate of being 1.2 years younger in our data.

**Table 2.** Association Between Green Space (per IQR, 270-m Buffer) and Cogstate *z* Scores, Nurses’ Health Study II

Measure	Green space exposure, β (95% CI) (N = 13 573)^a^	*P* value
Learning/working memory		
Age and race (basic)	0.02 (−0.01 to 0.04)	.14
Plus childhood factors	0.02 (−0.004 to 0.05)	.10
Plus adulthood socioeconomic indicators	0.02 (−0.004 to 0.05)	.09
Plus neighborhood socioeconomic status	0.03 (−0.0003 to 0.05)	.05
Psychomotor speed/attention		
Age and race (basic)	0.04 (0.02 to 0.07)	<.001
Plus childhood factors	0.04 (0.02 to 0.07)	<.001
Plus adulthood socioeconomic indicators	0.04 (0.02 to 0.07)	<.001
Plus neighborhood socioeconomic status	0.05 (0.02 to 0.08)	<.001
Global Cogstate		
Age and race (basic)	0.04 (0.01 to 0.06)	.002
Plus childhood factors	0.04 (0.02 to 0.06)	.001
Plus adulthood socioeconomic indicators	0.04 (0.02 to 0.07)	.001
Plus neighborhood socioeconomic status	0.05 (0.02 to 0.07)	<.001

^a^ Higher scores indicate better cognitive performance.

We observed positive nonlinear associations between green space and the psychomotor speed/attention composite as well as the global score, whereas a U-shaped association was observed for the learning/working memory composite, after accounting for further confounders (eFigure 2 in the Supplement). Increasing green space was associated with higher scores on the psychomotor speed/attention composite, although the evidence of consistent improvement at levels of green space above 0.8 NDVI was not clear (eFigure 2 in the Supplement). Although the test for deviations from linearity for green space was significant for both psychomotor speed/attention composite and the global Cogstate score, the associations were visually monotonic and, thus, we report the IQR results as the main findings. Moreover, the distribution of the residuals of the main models did not suggest deviation from normality.

### Stratified Analyses

We did not observe an association between green space and the learning/working memory composite among the different quartiles of Census tract median income and there was no statistical evidence of a difference in associations (*P* for interaction = .65). In contrast, we observed associations between green space and the psychomotor speed/attention composite (β, 0.08; 95% CI, 0.03-0.13) and the global score (β, 0.06; 95% CI, 0.01-0.11) among participants with higher Census tract median income (Table 3). We also observed variability in the psychomotor speed/attention composite across levels of population density, although there was no evidence of a significant difference (*P* for interaction = .51).

**Table 3.** Association Between Green Space (IQR, 270-m Buffer) and Cogstate *z* Scores Stratified by US Census Tract Median Income and Population Density

Variable	β (95% CI)^a^					*P* value for interaction
	First quartile:<$60 781	Second quartile: $60 784-$77 386	Third quartile: $77 391-$99 427	Fourth quartile: $99 444-$250 000		
Census-tract median income, USD^b^						
No.	3332	3406	3417		3429	
Learning/working memory	0.05 (−0.001 to 0.10)	0.03 (−0.03 to 0.08)	−0.004 (−0.05 to 0.05)		0.01 (−0.04 to 0.06)	.65
Psychomotor speed/attention	0.03 (−0.02 to 0.08)	0.06 (0.01 to 0.11)	0.01 (−0.04 to 0.06)		0.08 (0.03 to 0.13)	.50
Global Cogstate	0.04 (−0.006 to 0.09)	0.06 (0.002 to 0.11)	0.002 (−0.05 to 0.05)		0.06 (0.01 to 0.11)	.83
Population density, people/sq km^c^	First quartile: <110	Second quartile: 111-522	Third quartile: 523-1245		Fourth quartile: 1246-69 555	
No.	3317	3396	3424		3447	
Learning/working memory	0.002 (−0.05 to 0.06)	0.06 (0.01 to 0.12)	0.04 (−0.01 to 0.10)		−0.02 (−0.07 to 0.04)	.52
Psychomotor speed/attention	0.02 (−0.04 to 0.07)	0.02 (−0.03 to 0.08)	0.09 (0.03 to 0.15)		0.05 (−0.01 to 0.10)	.51
Global Cogstate	0.01 (−0.04 to 0.07)	0.05 (−0.01 to 0.10)	0.08 (0.02 to 0.14)		0.02 (−0.04 to 0.08)	.43

^a^ Higher scores indicate better cognitive performance.

^b^ Models were adjusted for age, race, childhood factors, adulthood socioeconomic factors, and depression.

^c^ Models were adjusted for age, race, childhood factors, adulthood socioeconomic factors, neighborhood socioeconomic status, and depression.

### Mediation

Estimates of the proportion of the association between green space and cognitive function that might be mediated by other factors (assuming that underlying assumptions of the mediation analyses hold) were significant for depression (Table 4). Depression was estimated to explain 3.95% (95% CI, 0.35%-7.55%) of the association between green space and psychomotor speed/attention and 6.30% (95% CI, 0.77%-11.81%) of the association between green space and overall cognition. The results did not suggest mediation of the association between green space and cognition by any other of the investigated mediators.

**Table 4.** Estimated Proportion of Association Between Green Space (IQR; 270-m buffer) and Cognition Explained by Air Pollution, Depression, and Physical Activity

Mediators at early childhood^a^^,^^b^	Estimate (95% CI)		
	Learning/working memory	Psychomotor speed/attention	Global Cogstate
Air pollution (n = 13 573)			
Indirect effect	0.001 (−0.002 to 0.004)	−0.0001 (−0.003 to 0.003)	0.001 (−0.002 to 0.003)
Direct effect	0.02 (−0.004 to 0.05)	0.05 (0.02 to 0.08)	0.04 (0.02 to 0.07)
Total effect	0.02 (−0.002 to 0.05)	0.05 (0.02 to 0.08)	0.05 (0.02 to 0.07)
Percentage mediated	4.58 (−7.91 to 17.07)	−0.01 (−5.69 to 5.66)	1.29 (−4.82 to 7.40)
Depression (n = 13 584)			
Indirect effect	0.003 (0.001 to 0.005)	0.002 (0.0005 to 0.003)	0.003 (0.001 to 0.005)
Direct effect	0.02 (−0.005 to 0.05)	0.05 (0.02 to 0.07)	0.04 (0.02 to 0.07)
Total effect	0.02 (−0.002 to 0.05)	0.05 (0.02 to 0.07)	0.05 (0.02 to 0.07)
Percentage mediated	11.94 (−2.72 to 26.59)	3.95 (0.35 to 7.55)	6.30 (0.77 to 11.81)
Physical Activity (n = 13 584)			
Indirect effect	0.001 (−0.00004 to 0.002)	0.001 (−0.0001 to 0.002)	0.001 (0.00001 to 0.002)
Direct effect	0.02 (−0.002 to 0.05)	0.05 (0.02 to 0.07)	0.04 (0.02 to 0.07)
Total effect	0.02 (−0.002 to 0.05)	0.05 (0.02 to 0.07)	0.05 (0.02 to 0.07)
Percentage mediated	3.50 (−1.69 to 8.69)	1.51 (−0.47 to 3.49)	2.09 (−0.30 to 4.48)

^a^ Mediation analyses assume that there is no unmeasured exposure-outcome confounding, no unmeasured mediator-outcome confounding, no unmeasured exposure-mediator confounding, and no mediator-outcome confounder affected by exposure.

^b^ Analytical sample for mediation analysis excluded participants with missing data on any of the mediators of interest.

 In sensitivity analyses, where we address the fact that the mediators of interest could also be confounders, we observed that adjustment for depression, air pollution, and physical activity in the main models did not change the association between green space and cognition (eTable 2 in the Supplement).

## Discussion

This nationwide cohort study of adult women investigated the association of residential surrounding green space in 270-m and 1230-m buffers with cognitive function. Our study found that higher levels of residential green space were associated with higher scores on processing speed and attention and on overall cognition, after adjusting for age, race, individual childhood and adulthood SES, and neighborhood SES. The estimate of the association between green space and processing speed and attention was equivalent to the estimate of being 1.2 years younger, in our data. We also found that higher levels of residential green space were not associated with learning/working memory battery scores. Our estimates were robust to a wide range of sensitivity analyses.

Our results were similar to those of a 10-year longitudinal study^6^ of British civil servants from the Whitehall cohort that found that an IQR increase in NDVI was associated with a difference in the global cognition *z* score (0.02; 95% CI, 0.003-0.037) in the 500-m buffer. Our results were also consistent with a recent analysis^48^ of US Medicare beneficiaries aged less than 65 years that suggested that higher green space was associated with reduced risk of Alzheimer Disease, after adjustment for individual and neighborhood sociodemographics.

The associations with psychomotor speed/attention were greater among participants living in neighborhoods with higher SES. This contrasted with the Whitehall study,^6^ which found no clear trends in the associations with cognitive function across strata of neighborhood SES. This difference might be due to different distributions and variation of neighborhood SES among the US and the English populations. A recent article^49^ reported that the economic inequalities in the UK are substantially lower compared with inequality in the USA. Our results also contrast with those by Cherrie et al,^7^ who found an association between green space and cognition among lower SES groups. However, the latter study used parental occupational social class as a measure of SES from childhood, whereas we used neighborhood SES. All our participants were nurses at baseline so there was likely less variation in SES, which might explain the difference in our results.

The null asociations between green space and learning and working memory are in accordance with a recent study^50^ that examined the association between green space and brain-based magnetic resonance imaging measures in older adults. The authors^50^ found that living in neighborhoods with more green space was not associated with left or right hippocampal volume, which is responsible for memory and learning. In line with previous research, this study suggests differing associations between green space and cognition based on the cognitive domain examined.^51,52^

Previous research on green space and cognition has reported greater associations for women compared with men,^6,7^ which may explain the robust associations found in our study, despite the fact that the women in our study were younger than those in previous studies, which might be expected to reduce our ability to detect associations. However, this may also signal the public health importance of green space, if associations were detected in these young-older women. Furthermore, subtle alterations in continuous measures of cognition have important clinical implications at the population level.^53^

We evaluated several potential mediatiors on the association between green space and cognitive function. Higher green space exposure has been associated with lower exposure to air pollution, lower risk of depression, and increased opportunities for physical activity,^40,54^ which, in turn, could improve cognition. However, in our study we did not find evidence that the association between green space and cognitive function could be explained by air pollution or physical activity. Our results on physical activity and air pollution are in agreement with a previous study^6^ that evaluated these as potential mediators between green space and cognitive decline. Our mediation analysis did suggest that green space may be associated with cognitive function through depression. There is a foundation for this mechanism in the literature. Higher exposure to green space has been consistently linked to lower levels of depression.^55,56^ In addition, depression has been documented to be an important factor associated with risk for dementia.^41,57^ These findings highlight the relevance of green space as a potential factor to reduce depressive episodes and thus subsequent dementia.

### Limitations and Strengths

Potential limitations of this study include the outcome measurement at a single time point, which did not allow us to examine causal associations or temporality of associations. However, the Cogstate battery was administered at least 1 year after the green space exposure measure. Further longitudinal studies that examine associations between exposure to green space and cognitive decline would be informative. Second, our study population was predominantly White women with high education levels. This limits examination of racial disparities, effect modification by SES, or generalizability to populations with substantially different distributions of green space. In addition, the most appropriate buffer within which to measure green space is not clear.^58^ We examined buffers around the immediate area of residences (270 m) and walkable area (1230 m) as potential relevant geographic contexts. In both analyses, we observed similar results, but it is unclear whether these associations would remain if other buffer sizes were examined or if we were able to incorporate information about where participants spent their time when not at home.^59^ Third, NDVI does not measure the quality of green space or green space features as individuals experience them. Future studies should look into green space quality and ground-level metrics that can capture how individuals experience their environment, including street-view imagery or smartphone Global Positioning System data. Fourth, the assumption of temporal ordering of exposure, mediators, and outcome may not be satisfied, nor would the no unmeasured confounding assumption, particularly for the exposure-mediator as they were both measured at the same time.

Our study also has important strengths. We accounted for multiple individual childhood and adulthood socioeconomic factors and neighborhood SES as potential confounders. In addition, we evaluated several domains of cognition through the Cogstate battery, which is an objective measure of cognitive function and has been shown to be a sensitive indicator of early cognitive deficits.^32^ This is among the first examinations to study the association between green space exposure and cognitive function across the entire US. Finally, measurements of important environmental exposures, mental health, and behaviors enabled us to examine potential mechanisms through which green space is associated with cognitive function.

## Conclusions

We observed that higher residential surrounding green space was associated with better processing speed and attention, as well as overall cognition among participants in the Nurses’ Health Study II. The worldwide aging population and the rapid increase of dementia calls for novel prevention strategies. Our results suggest that green space exposure should be investigated as a potential population-level approach to improve cognitive function.

## Notes

Supplement. **eAppendix.** Supplemental Methods **eTable 1.** Association of Green Space Exposure (per IQR) in the 1230m Buffer Surrounding Each Address With Cogstate Z-scores in Middle Age, Nurses' Health Study II, 2014-2016 **eFigure 1.** Direct and Indirect Associations Between Green Space and Cognitive Decline **eFigure 2.** Nonlinear Associations Between Green Space in the 270m Buffer Surrounding Each Address and (A) Learning/Working Memory, (B) Psychomotor Speed/Attention, and (C) Global Cogstate in Middle Age, Nurses' Health Study II, 2014-2016 **eTable 2.** Association of Green Space Exposure in a 270m Buffer Around Each Address (per IQR) With Cogstate Z-Scores in Middle Age, Nurses' Health Study II, 2014-2016, Further Adjusted for Air Pollution, Depression, and Physical Activity
eReferences
