# Supplementary material for: Residential Green Space and Cognitive Function in a Large Cohort of Middle-Aged Women
Source: JAMA Netw Open. 2022 Apr 27;5(4):e229306. doi: 10.1001/jamanetworkopen.2022.9306 (PMC9047638; doi:10.1001/jamanetworkopen.2022.9306)
Supplement: Supplement. — eAppendix. Supplemental Methods eTable 1. Association of Green Space Exposure (per IQR) in the 1230m Buffer Surrounding Each Address With Cogstate Z-scores in Middle Age, Nurses' Health Study II, 2014-2016 eFigure 1. Direct and Indirect Associations Between Green Space and Cognitive Decline eFigure 2. Nonlinear Associations Between Green Space in the 270m Buffer Surrounding Each Address and (A) Learning/Working Memory, (B) Psychomotor Speed/Attention, and (C) Global Cogstate in Middle Age, Nurses' Health Study II, 2014-2016 eTable 2. Association of Green Space Exposure in a 270m Buffer Around Each Address (per IQR) With Cogstate Z-Scores in Middle Age, Nurses' Health Study II, 2014-2016, Further Adjusted for Air Pollution, Depression, and Physical Activity eReferences [file jamanetwopen-e229306-s001.pdf]

Supplemental Online Content

Jimenez MP, Elliott EG, DeVille NV, et al. Residential green space and cognitive function in a large cohort of middle-aged women. *JAMA Netw Open*. 2022;5(4):e229306. doi:10.1001/jamanetworkopen.2022.9306

eAppendix. Supplemental Methods

eTable 1. Association of Green Space Exposure (per IQR) in the 1230m Buffer Surrounding Each Address With Cogstate Z-scores in Middle Age, Nurses' Health Study II, 2014-2016

eFigure 1. Direct and Indirect Associations Between Green Space and Cognitive Decline

eFigure 2. Nonlinear Associations Between Green Space in the 270m Buffer Surrounding Each Address and (A) Learning/Working Memory, (B) Psychomotor Speed/Attention, and (C) Global Cogstate in Middle Age, Nurses' Health Study II, 2014-2016

eTable 2. Association of Green Space Exposure in a 270m Buffer Around Each Address (per IQR) With Cogstate Z-Scores in Middle Age, Nurses' Health Study II, 2014-2016, Further Adjusted for Air Pollution, Depression, and Physical Activity

eReferences

This supplemental material has been provided by the authors to give readers additional information about their work.

© 2022 Jimenez MP et al. *JAMA Network Open.*

eAppendix. Supplemental Methods

Green space assessment

NDVI is the most widely used satellite-derived indicator of the quantity of vegetation on the ground and has been used as a marker for exposure to green space in previous epidemiological studies,[1,2] including brain health studies,[3] and within the Nurses' Health Study cohorts.[4] We used NDVI from July 2013, since NDVI reaches its maximum and highest level of geographic variation during the height of the summer. We used Landsat 8 satellite data at 30m resolution and 2013 NDVI to represent long-term exposure.

Cognitive Assessment

DET requires the respondent to press the "Yes" key as soon as a playing card on the screen is turned face up, and measures processing speed and psychomotor function. IDN measures attention; when the card flips over the respondent indicates "yes" if the card is red. OCL measures visual learning; the card flips over and the respondent responds "yes" if the same card has been seen before in the test. Finally, ONB measures working memory; the card flips over and the respondent presses "yes" if it is identical to the one presented just before it.
Cogstate has been found to predict early Alzheimer's dementia pathology in non-cognitively impaired persons.[5] Moreover, Cogstate has been used to identify risk factor associations previously in this cohort.[6,7]

Air pollution assessment

Air pollution was predicted from a spatiotemporal generalized additive mixed model that predicts monthly outdoor concentrations of particulate matter with an aerodynamic diameter less than 2.5 microns ($PM_{2.5}$) at each residential address and was quantified as the twenty-four month moving averages for each questionnaire cycle between 2013-2015 as a measure of long-term exposures .[8]

Mediation Analysis

Following Valeri and Vanderweele,[9] we fitted a parametric linear regression model for cognitive function (continuous) and a parametric regression model for the mediator (continuous), and computed them analytically. Under the assumptions that there are no unmeasured exposure-outcome confounders, no unmeasured mediator-outcome confounders, no unmeasured exposure-mediator confounders and no mediator-outcome confounder affected by exposure, and provided our models are correctly specified, the combined estimates from the two models provided the controlled direct effect, natural direct effect, and natural indirect effect.

© 2022 Jimenez MP et al. *JAMA Network Open.*

eTable 1. Association of Green Space Exposure (per IQR) in the 1230m Buffer Surrounding Each Address With Cogstate Z-scores in Middle Age, Nurses' Health Study II, 2014-2016.

	**Green Space Exposure**		
**Learning/working memory**			
	N	**β (95% CI)**	**p-value**
Age and race (basic)	13573	0.01 (-0.01, 0.03)	0.33
+ childhood factors	13573	0.01 (-0.01, 0.03)	0.22
+ adulthood socioeconomic indicators	13573	0.01 (-0.01, 0.03)	0.21
+ neighborhood socioeconomic status	13573	0.02 (-0.01, 0.04)	0.12
**Psychomotor speed/attention**			
		**β (95% CI)**	**p-value**
Age and race (basic)	13573	0.04 (0.01, 0.06)	<0.001
+ childhood factors	13573	0.04 (0.02, 0.06)	<0.001
+ adulthood socioeconomic indicators	13573	0.04 (0.02, 0.06)	<0.001
+ neighborhood socioeconomic status	13573	0.04 (0.02, 0.07)	<0.001
**Global Cogstate**			
		**β (95% CI)**	**p-value**
Age and race (basic)	13573	0.03 (0.01, 0.05)	<0.01
+ childhood factors	13573	0.03 (0.01, 0.05)	<0.01
+ adulthood socioeconomic indicators	13573	0.03 (0.01, 0.05)	<0.01
+ neighborhood socioeconomic status	13573	0.04 (0.02, 0.06)	<0.01

© 2022 Jimenez MP et al. *JAMA Network Open.*

eFigure 1. Direct and Indirect Associations Between Green Space and Cognitive Decline.

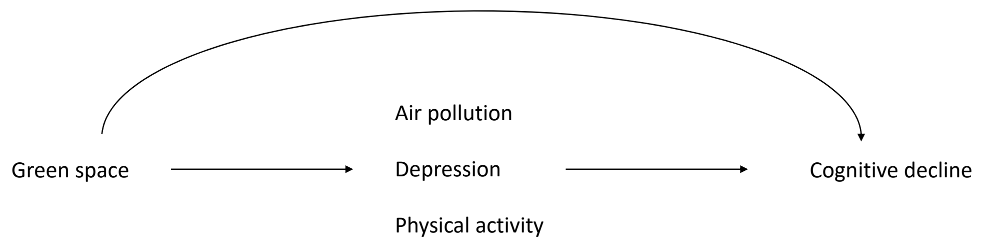

© 2022 Jimenez MP et al. *JAMA Network Open.*

eFigure 2. Nonlinear Associations Between Green Space in the 270m Buffer Surrounding Each Address and (A) Learning/Working Memory, (B) Psychomotor Speed/Attention, and (C) Global Cogstate in Middle Age, Nurses' Health Study II, 2014-2016.[a]

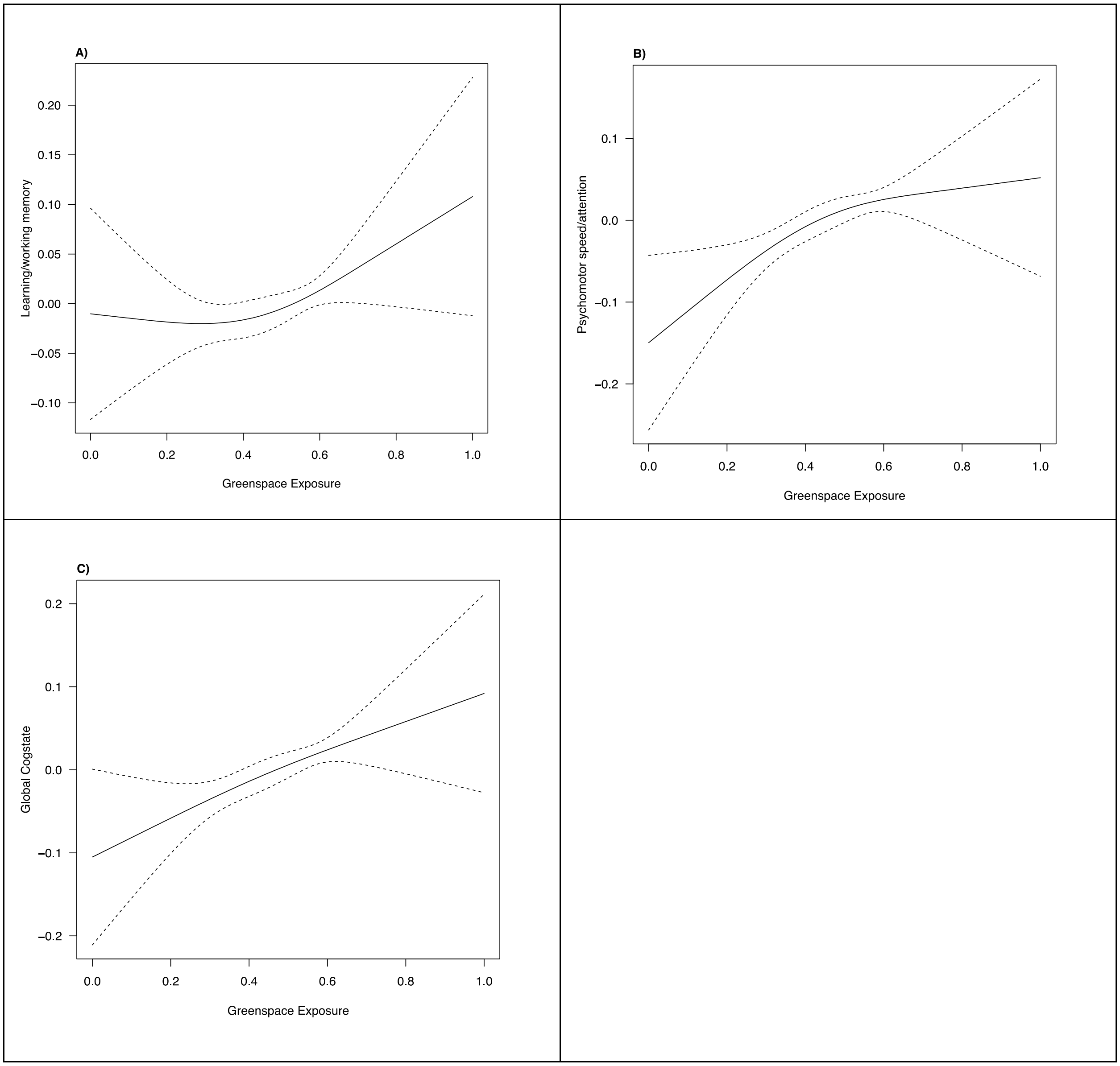

[a] P-values correspond to spline terms for greenness exposure at each time point: A) p=0.11, B) p<0.01, C) p= p<0.01

© 2022 Jimenez MP et al. *JAMA Network Open.*

eTable 2. Association of Green Space Exposure in a 270m Buffer Around Each Address (per IQR) With Cogstate Z-Scores in Middle Age, Nurses' Health Study II, 2014-2016, Further Adjusted for Air Pollution, Depression, and Physical Activity.[a]

**Learning/working memory**			
Further adjusted by:	N	**β (95% CI) for green space estimate**	**p-value**
Air pollution	13573	0.03 (-0.002, 0.05)	0.07
Depression	13573	0.02 (-0.003, 0.05)	0.09
Physical Activity	13573	0.03 (-0.001, 0.05)	0.06
**Psychomotor speed/attention**			
Further adjusted by:		**β (95% CI) for green space estimate**	**p-value**
Air pollution	13573	0.05 (0.02, 0.08)	<0.001
Depression	13573	0.05 (0.02, 0.08)	<0.001
Physical Activity	13573	0.05 (0.02, 0.08)	<0.001
**Global Cogstate**			
Further adjusted by:		**β (95% CI) for green space estimate**	**p-value**
Air pollution	13573	0.05 (0.02, 0.07)	<0.001
Depression	13573	0.04 (0.02, 0.07)	0.001
Physical Activity	13573	0.05 (0.02, 0.07)	<0.001

[a] All models adjusted for age, race, childhood factors, adulthood socioeconomic status, and neighborhood socioeconomic status.

© 2022 Jimenez MP et al. *JAMA Network Open.*

eReferences

1. James P, Banay RF, Hart JE, Laden F. A Review of the Health Benefits of Greenness. *Curr Epidemiol Rep*. 2015;2(2):131-142. doi:10.1007/s40471-015-0043-7

2. Fong K, Hart JE, James P. A Review of Epidemiologic Studies on Greenness and Health: Updated Literature Through 2017. *Curr Environ Health Rep*. 2018;5:77-87. doi:10.1007/s40572-018-0179-y.A

3. Besser L. Outdoor green space exposure and brain health measures related to Alzheimer's disease: a rapid review. *BMJ Open*. 2021;11(5):e043456. doi:10.1136/bmjopen-2020-043456

4. James P, Berrigan D, Hart JE, et al. Effects of buffer size and shape on associations between the built environment and energy balance. *Health Place*. 2014;27:162-170. doi:10.1016/j.healthplace.2014.02.003

5. Darby DG, Brodtmann A, Pietrzak RH, et al. Episodic memory decline predicts cortical amyloid status in community-dwelling older adults. *J Alzheimers Dis*. 2011;27(3):627-637. doi:10.3233/JAD-2011-110818

6. Sumner JA, Hagan K, Grodstein F, Roberts AL, Harel B, Koenen KC. Posttraumatic stress disorder symptoms and cognitive function in a large cohort of middle-aged women. *Depress Anxiety*. 2017;34(4):356-366. doi:10.1002/da.22600

7. Roberts AL, Sumner JA, Koenen KC, et al. Childhood Abuse and Cognitive Function in a Large Cohort of Middle-Aged Women. *Child Maltreat*. Published online November 9, 2020:1077559520970647. doi:10.1177/1077559520970647

8. Yanosky JD, Paciorek CJ, Laden F, et al. Spatio-temporal modeling of particulate air pollution in the conterminous United States using geographic and meteorological predictors. *Environmental Health*. 2014;13(1):63. doi:10.1186/1476-069x-13-63

9. Valeri L, Vanderweele TJ. Mediation analysis allowing for exposure-mediator interactions and causal interpretation: theoretical assumptions and implementation with SAS and SPSS macros. *Psychol Methods*. 2013;18(2):137-150. doi:10.1037/a0031034

© 2022 Jimenez MP et al. *JAMA Network Open.*
